# Caveolin-1 is involved in fatty infiltration and bone-tendon healing of rotator cuff tear

**DOI:** 10.1186/s10020-023-00627-4

**Published:** 2023-03-14

**Authors:** Shanhong Fang, Mengqiang You, Jie Wei, Peng Chen

**Affiliations:** 1 Department of Sports Medicine, National Regional Medical Center, Binhai Campus of the First Affiliated Hospital of Fujian Medical University, Fuzhou, 350212 China; 2 Department of Orthopedic Surgery, The First Affiliated Hospital of Fujian Medical University, No. 20, Chazhong Road, Taijiang District, Fujian Province 350005 Fuzhou, China

**Keywords:** Rotator cuff tear, Bone-tendon healing, Fatty infiltration, Caveolin-1, GATA6, cAMP/PKA pathway

## Abstract

**Background:**

Caveolin-1 has been predicted, based on RNA transcriptome sequencing, as a key gene in rotator cuff tear (RCT) and it is related to fatty infiltration. This study aims to elucidate the upstream and downstream mechanism of Caveolin-1 in fatty infiltration and bone-tendon healing after RCT in rat models.

**Methods:**

Differentially expressed genes related to RCT were screened, followed by functional enrichment analysis and protein-protein interaction analysis. GATA6 was overexpressed and Caveolin-1 was knocked down in tendon stem cells (TSCs) to evaluate their effects on the adipogenic differentiation of TSCs. In addition, a RCT rat model was constructed and injected with lentivirus carrying oe-GATA6, oe-Caveolin-1 alone or in combination to assess their roles in fatty infiltration and bone-tendon healing.

**Results and conclusion:**

Caveolin-1 was identified as a key gene involved in the RCT process. In vitro results demonstrated that Caveolin-1 knockdown inhibited adipogenic differentiation of TSCs by activating the cAMP/PKA pathway. GATA6 inhibited the transcription of Caveolin-1 and inhibited its expression, thus suppressing the adipogenic differentiation of TSCs. In vivo data confirmed that GATA6 overexpression activated the cAMP/PKA pathway by downregulating Caveolin-1 and consequently repressed fatty infiltration, promoted bone-tendon healing, improved biomechanical properties and reduced the rupture risk of injured tendon in rats after RCT. Overall, this study provides novel insights into the mechanistic action of Caveolin-1 in the fatty infiltration and bone-tendon healing after RCT.

**Supplementary Information:**

The online version contains supplementary material available at 10.1186/s10020-023-00627-4.

## Introduction

Tendon repair is a frequently used treatment method for the small and medium-sized rotator cuff tears (RCTs) (Moosmayer et al. 2019). Most RCTs occur at the bone-tendon interface and cause disability and pain (Saveh-Shemshaki et al. 2019, Lin et al. 2021). Despite great advances in surgical techniques for RCT, the postoperative failure of RCT is still high due to the poor healing competence of bone-tendon interface (Tong et al. 2023). Fatty infiltration after tendon injury is a very common event and compromises the outcomes of tendon injury healing process (Wang et al. 2019). Therefore, exploring the specific mechanism of fatty infiltration after RCT is of great value for promoting bone-tendon healing.

GATA binding protein 6 (GATA6) is a member of the GATA family of zinc-finger transcriptional regulators, which plays key roles in the development of several mesodermally derived cell lineages (Morrisey et al. 1996). GATA members have participated in the healing of bone fractures (Kacena et al. 2013; Liao et al. 2017). The involvement of GATA6 in the bone-tendon healing remains to be elucidated. GATA6 exhibits an inverse correlation with the expression of Caveolin-1 in mouse models of asthma and silencing of GATA6 upregulates Caveolin-1 expression (Fang et al. 2016). Caveolin-1 is known as an oncogenic membrane protein related to endocytosis, extracellular matrix organization, cholesterol distribution, cell migration and signaling (Nwosu et al. 2016). Caveolin-1 can function as a slow-release material to accelerate bone-tendon junction healing via promoting the formation of the transition zone (Liang et al. 2019).

In addition, silencing of Caveolin-1 contributes to promotion of the cAMP/PKA pathway activation (Kuo et al. 2018). The cAMP/PKA pathway has been involved in the regulation of adipogenic differentiation of tendon stem cells (TSCs) by IGF-1 and BMP-2 (Liu et al. 2014). TSCs are derived from tendon tissues and are the endogenous cell source for tenocyte turnover and tendon functional maintenance, playing a vital role in tendon repair and regeneration (Shi et al. 2017; Lai et al. 2021). We therefore hypothesized that GATA6 could affect the fatty infiltration and bone-tendon healing after RCT via regulation of the Caveolin-1/cAMP/ PKA axis. The present study was designed to evaluate the role of GATA6 in RCT in a rat model and provide a new light on the mechanism underlying the fatty infiltration and bone-tendon healing after RCT.

## Materials and methods

### Transcriptome RNA sequencing data source

The RNA sequencing dataset GSE103266 (12 RCT rat supraspinatus tendon tissue samples and 4 control tissue samples) was downloaded from the Gene Expression Omnibus (GEO) database. RCT rats underwent bilateral full-thickness supraspinatus tendon tear and suprascapular nerve resection, and samples were collected at 10, 30 and 60 days after surgery, respectively (n = 4). These data have been made accessible in open-access databases and this study was thus not required to be approved by the ethics committee.

### Screening of differentially expressed genes (DEGs)

R “limma” package was applied for differential analysis of the GSE103266 dataset to identify the DEGs between RCT samples (10 days after surgery) and control samples (DEG1), RCT samples of 10 days after surgery and of 30 days after surgery (DEG2), RCT samples of 30 days after surgery and of 60 days after surgery (DEG3), with *p* value < 0.05 as the threshold. The jvenn tool was then used to intersect DEG1, DEG2, and DEG3.

### Functional enrichment analysis

The R “clusterProfiler” software package was performed for GO and KEGG enrichment analysis of DEGs with *p* value < 0.05 as the threshold. Genes enriched in the top five GO entries were intersected with those enriched in the top five KEGG pathways using the jvenn tool and the obtained candidates were used for subsequent Protein-protein interaction analysis.

### Protein–protein interaction analysis

The interaction between the proteins encoded by the intersected genes was analyzed by the STRING website, with the Degree value representing the number of proteins connecting with the other proteins. Based on the Degree value, ranking was conducted.

### Isolation, identification and culture of tendon stem cells (TSCs)

Twelve-week-old SD rats (weighing: 250–300 g; purchased from Hunan SJA Laboratory Animal Co., Ltd., Hunan, China) were included in this study. The patellar tendon of the rat knee joint was collected after which the tendon sheath and the surrounding anterior patellar muscle were carefully removed. The tendon tissue was cut into small pieces and then digested using 0.2% type I collagenase (SCR103, Sigma) in low-glucose DMEM (D5030, Sigma) for 1 h at 37 °C. The digested cells were suspended in low-glucose DMEM supplemented with 10% FBS, 100 U/mL penicillin, and 100 mg/mL streptomycin, with the medium changed every 3 days.

TSC surface-specific markers were determined by flow cytometry. Cells at passage 2 were incubated with FITC-labeled monoclonal antibodies to CD44 (ab244581, 0.2 µg/mL, Abcam, Cambridge, UK), CD90 (ab226, 1: 500, Abcam), CD34 (ab81289, 1:50, Abcam), CD45 (ab6329, 02 µg/mL, Abcam), and CD146 (ab75769, 1:50, Abcam) for 30 min, with FITC-labeled IgG (A0556, Beyotime, Shanghai, China) set as isotype control. Next, cells were resuspended in 10% normal goat serum and analyzed with CyAn ADP analyzer (Beckman Coulter Inc., Chaska, MN, USA).

Next, adipogenic, osteogenic, and chondrogenic differentiation potential of TSCs were determined in cells at passage 2 using oil red O staining, and Alizarin red S (ARS) staining, respectively (Li et al. 2018; Wang et al. 2019).

### Cell treatment

Upon reaching 20–30% confluence, TSCs seeded in 6-well plates (8 × 10^4^ cells/well) were transfected using Lipofectamine 2000 reagent (11668019, Thermo Fisher Scientific Inc., Waltham, MA, USA) with plasmids of sh-NC, sh-Caveolin-1#1, sh-Caveolin-1#2, sh-Caveolin-1#3, oe-NC, oe-GATA6, oe-NC + sh-NC, oe-GATA6 + sh-NC, and oe-GATA6 + sh-Caveolin-1 or treated with sh-Caveolin-1 + DMSO, and sh-Caveolin-1 + H89 (PKA inhibitor; 10 µM for 24 h; B1427; Sigma; dissolved in DMSO). The shRNA sequences are shown in Additional file 2: Table S1.

Gene overexpression plasmid pCMV6-AC-GFP was purchased from Shanghai Yaji Biological Technology Co., Ltd. (Shanghai, China; YC-13849RJ) and gene knockdown plasmids from Thermo Fisher Scientific. After 48 h, transfection efficiency was determined by RT-qPCR.

### ChIP assay

ChIP assay was conducted as previously described (Zhu et al. 2016). Briefly, cells were fixed with 1% formaldehyde at room temperature for 10 min and lysed. The genome was sonicated to fragments of appropriate size. Positive control RNA polymerase II rabbit antibody (ab238146, 1: 100, Abcam), NC rabbit anti-IgG (ab172730, 1:100, Abcam) and target protein-specific antibody to GATA6 (rabbit, SAB2100899, 1 mg/mL, Thermo Fisher Scientific) were added into the lysates and incubated overnight at 4 °C, followed by incubation with Protein Agarose/Sepharose. Finally, precipitants were analyzed by qPCR. Specific primers of Caveolin-1 gene promoters are forward: CAATAAGACAAAGGAGGCTG and reverse: TGTGCTTGACTGTGAGAGAAT.

### Dual-luciferase reporter assay

Dual-luciferase reporter plasmids PGLO-Caveolin-1 WT and PGLO-Caveolin-1 MUT (Caveolin-1 WT: GAAAGAAAAGAAA; Caveolin-1 MUT: GUUACAGGTAUGA) were co-transfected with the plasmids of oe-GATA6 and oe-NC into HEK-293T cells (iCell-h237, iCell Bioscience Inc., Shanghai, China) for 24 h. As normalized to Renilla luciferase, relative luciferase activity was determined using Dual-Luciferase^®^ Reporter Assay System (E1910, Promega, Madison, WI, USA) (Liu et al. 2018).

### Construction of rat models

Fifty-six rats were maintained in specific pathogen-free conditions with 60% humidity at 25 °C under a 12-h light/dark cycle with ad libitum access to standard food and water, were used in this study. A supraspinatus tear model was induced as previously described (Oak et al. 2014). During postoperative recovery, rats received subcutaneous 0.05 mg/kg buprenorphine for analgesia. Rats were allowed with free load and cage movement, and monitored for signs of distress or infection. All rats had significant ability to walk to the same extent and showed signs of adequate food and water intake.

After surgical repair of the torn supraspinatus tendon, rats were injected with 2 µL lentivirus (9 × 10^8^ TU/mL; Shanghai Genechem Co., Ltd., Shanghai, China) carrying sh-NC, sh-Caveolin-1, oe-NC, oe-GATA6 + oe-NC, and oe-GATA6 + oe-Caveolin-1 or treated with oe-GATA6 + oe-Caveolin-1 + forskolin (10 mg/kg; cAMP/PKA pathway activator; F6886, Sigma) under the acromion, once daily for 2 weeks. Sham rats (injection with normal saline) were regarded as the control, with eight rats for each treatment.

After tissue resection, the rats were euthanized with an overdose of pentobarbital sodium. The distal two-thirds plus the tendon and humeral head were used for measurements of mechanical properties. For the right shoulder, approximately two-thirds were minced and used for gene expression analysis while the distal third of the muscle and humeral head with the enthesis were used for histology. All assays were performed in a blinded fashion.

### Masson’s trichrome staining

Paraffin sections were heated, dewaxed, dehydrated and soaked in 10% trichloroacetic acid and 10% potassium dichromate for 40 min, respectively. Next, the sections were stained with hematoxylin (PT001, Shanghai Bogu Biotechnology Co., Ltd., Shanghai, China) for 8 min, and counterstained in a mixture of 1% ponceau S solution (R21983, Saint Biotechnology, Shanghai, China) with 1% fuchsin (R23166, Saint Biotechnology) for 40 min. The reaction was terminated with 1% glacial acetic acid, and 1% molybdic acid and then with a mixture of 1% bright green, 1% phosphomolybdic acid and tap water. Finally, the sections were dehydrated, cleared, mounted and observed under an optical microscope (BX50, Olympus Corp, Tokyo, Japan). The continuity, parallel orientation, density, and maturity of collagen fibers, as well as the number of blood vessels and cells were assessed by three blinded and semi-quantitative methods based on a previous study (Yoon et al. 2020).

### Immunohistochemistry

After antigen retrieval in water bath and normal goat serum (C0265, Beyotime) blocking, the slides were incubated with primary antibodies to Caveolin-1 (ab32577, 1:2000, Abcam) or collagen I (ab270993, 1:500, Abcam) (Fang et al. 2018), which was followed by incubation with secondary antibody goat anti-rabbit IgG (ab6721, 1:1000, Abcam). Finally, the staining processes were performed with diaminobenzidine colorimetric reagent solution (P0203, Beyotime) and hematoxylin (C0107, Beyotime). HE staining was used for histological observation as previously described (Liu et al. 2021).

### Biomechanical test

The Achilles tendon with the upper and lower bone ends was isolated. The two bone ends were fixed to a custom test fixture with two clamps, with the calcaneus end fixed at the lower clamp and the tibial end to the upper clamp. The mechanical test machine was then connected to the computer test system. The operational parameters were entered after which the biomechanical test was conducted, with six to eight samples used per group.

### RT-qPCR

Total RNA was extracted from tissues and cells using TRIzol (16096020, Thermo Fisher Scientific), the concentration and purity of which were determined using a spectrophotometer (1.8 ≤ A260/A280 ratio ≤ 2.0). The RNA was reverse transcribed into cDNA using cDNA RT reagent Kit (RR047A, Takara, Japan). The synthesized cDNA was subjected to RT-qPCR detection with the LightCycler 480 SYBR Green I Master (04707516001, Roche, Germany). The relative expression of mRNA was analyzed using the 2^-ΔΔCt^ method, with GAPDH as the internal reference. All primer sequences are shown in Additional file 2: Table S2.

### Western blot

Protein lysates were generated using RIPA buffer with PMSF (P0013B, Beyotime) and protein concentrations quantified by BCA Protein Assay (P0028, Beyotime). Lysates were loaded on 8–12% SDS-PAGE gels and transferred onto PVDF membranes (1620177, Bio-Rad Laboratories, Hercules, CA, USA). After 5% BSA blocking, the membrane was probed with primary antibodies to GATA6 (SAB2100899, 1 mg/mL, Thermo Fisher Scientific), Caveolin-1 (ab32577, 1:1000, Abcam), PKA (SAB4502337, 1 mg/mL, Thermo Fisher Scientific), pPKA (SAB4503969, phosphorylation site of Thr197, 1 mg /mL, Thermo Fisher Scientific), CREB (ab32515, 1:500, Abcam), pCREB (ab32096, phospho S133, 1:5000, Abcam), ATGL (ab207799, 1:1000, Abcam), pHSL (SAB4503879, phosphorylation site of Ser552, 1 mg/mL, Thermo Fisher Scientific), HSL (SAB4501762, 1 mg/mL, Thermo Fisher Scientific), FAS (ab82419, 1: 1000, Abcam), PPAR-γ (ab209350, 1:500, Abcam), pACC (ab68191, phospho S79, 1:5000, Abcam), ACC (ab45174, 1:1000, Abcam) and GAPDH (ab8245, 1:500, Abcam). Afterwards, the membrane was incubated with HRP-conjugated secondary antibody goat anti-rabbit IgG (ab6721, 1: 5000, Abcam) for 1 min at room temperature. ECL reagent (1705062, Bio-Rad) was used for visualization, with GAPDH used as an internal reference.

### Statistical analysis

Unpaired *t*-test, and one-way ANOVA with Tukey’s tests were utilized to calculate statistical significance for two-group and multi-group data. All results processed using SPSS 21.0 software were presented as mean ± SD. *p* < 0.05 suggests statistically significant difference.

## Results

### Caveolin-1 is a key gene involved in the RCT process

**Fig. 1:**
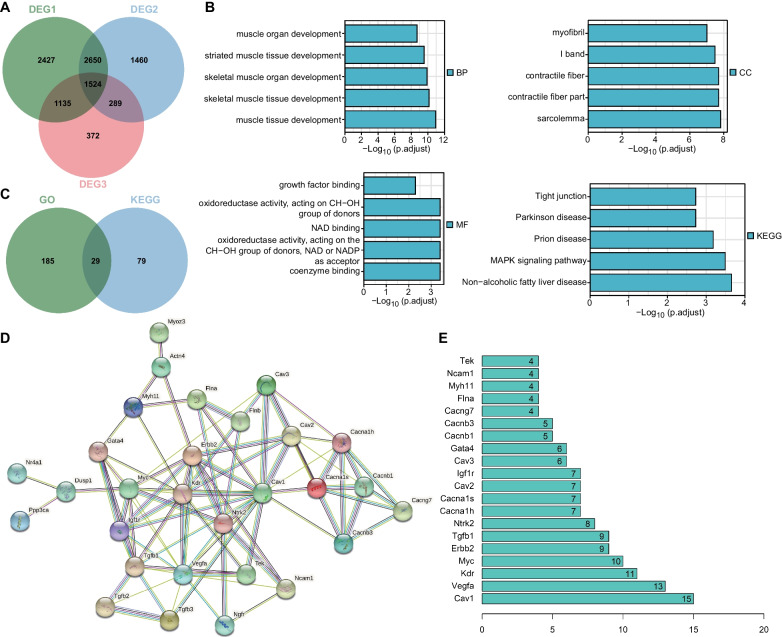
Bioinformatics analysis of key DEGs in RCT. **A** Venn diagram of DEG1, DEG2 and DEG3. **B** GO and KEGG enrichment analysis of 1524 intersected genes. The column length indicates *p* value, and the higher length reflects smaller *p* values. **C** Venn diagram of genes enriched in the main GO entries and KEGG pathway. **D** Protein–protein interaction analysis of proteins encoded by 29 intersected genes. **E** A ranking map of the top 20 genes based on the Degree value

Differential analysis of the transcriptome RNA sequencing dataset GSE103266 yielded 7736 DEG1, 5923 DEG2, and 3320 DEG3. Following intersection analysis of these genes identified 1524 candidate genes, namely genes with significant expression changes in the RCT process (Fig. 1A).

GO enrichment analysis suggested that these 1524 candidate genes were mainly enriched in BP entries of muscle tissue development, skeletal muscle tissue development, skeletal muscle organ development, and striated muscle tissue development, CC entries of sarcolemma, contractile fiber part, contractile fiber, and I band and MF of coenzyme binding, oxidoreductase activity, acting on the CH-OH group of donors, NAD or NADP as acceptor, NAD binding, oxidoreductase activity, and acting on CH-OH group of donors. This suggests that these intersected genes may play an important role in the RCT process (Fig. 1B). In addition, KEGG enrichment analysis indicated that these genes dominated in Non-alcoholic fatty liver disease, MAPK signaling pathway, Prion disease, Parkinson disease, and Tight junction, with Non-alcoholic fatty liver disease ranking the first (Fig. 1B). Thus, fatty infiltration may play a key role in RCT process.

To further screen the key genes in the RCT, the genes enriched in the top five GO entries were intersected with those enriched in the top five KEGG pathways, with 29 genes determined (Fig. 1C). Protein-protein interaction analysis of the proteins encoded by the 29 genes using the STRING database, with human as the species, showed that Caveolin-1 ranked the first based on the Degree value (Fig. 1D, E).

Meanwhile, published literature has shown that Caveolin-1 secreted by adipose tissues and adipocytes can promote adipogenesis (Chang et al. 2017). In addition, Caveolin-1 plays a key functional and structural role in the regulation of lipid droplet biogenesis and metabolism in vivo (Cohen et al. 2004). Based on the above data, we speculated that Caveolin-1 may play a role in RCT process and is related to fatty infiltration.

### Supraspinatus tendon injury induces high expression of Caveolin-1

**Fig. 2:**
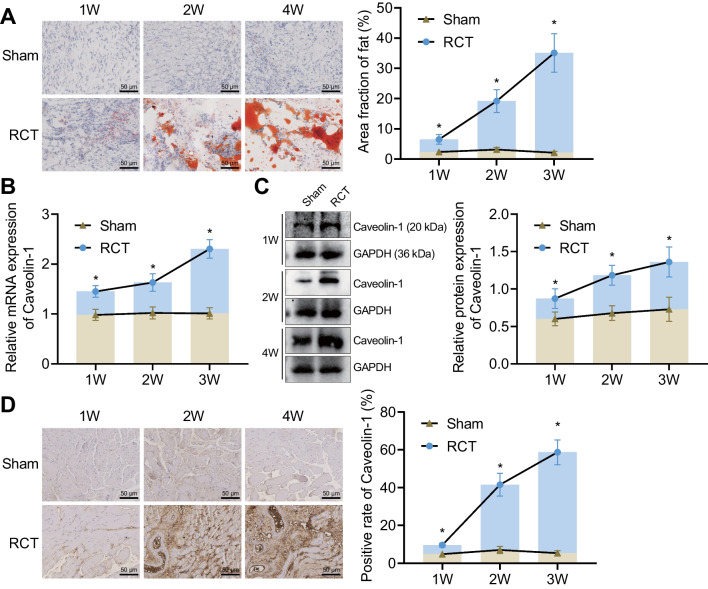
Abundant expression of Caveolin-1 in the supraspinatus tendon of RCT rat models. **A** Evaluation of fatty infiltration in the supraspinatus tendon of RCT rats at 1, 2 and 4 weeks after surgery. **B** Expression of Caveolin-1 in the supraspinatus tendon of RCT rats at 1, 2 and 4 weeks after surgery detected by RT-qPCR. **C** Western blot of Caveolin-1 protein in the supraspinatus tendon of RCT rats at 1, 2 and 4 weeks after surgery. **D** The positive expression of Caveolin-1 protein in the supraspinatus tendon of RCT rats at 1, 2 and 4 weeks after surgery. n = 8 rats for each treatment. *p < 0.05

Next, we constructed a bilateral supraspinatus tear-repair model and observed the fatty infiltration in the supraspinatus tendon and Caveolin-1 expression at 1, 2 and 4 weeks after surgery in rats. As shown in Fig. 2A, oil red O staining results showed increased fat area fraction in the supraspinatus tendon of RCT rats at 1, 2 and 4 weeks after surgery, with a progressive increase over time. The expression of Caveolin-1 was upregulated over time in the supraspinatus tendon of RCT rats at 1, 2 and 4 weeks after surgery, as revealed by RT-qPCR and Western blot (Fig. 2B, C). Meanwhile, the positive expression rate of Caveolin-1 was elevated in the supraspinatus tendon of RCT rats at 1, 2 and 4 weeks after surgery, mainly expressed in the fat region (Fig. 2D). The above results indicate that rat RCT can induce Caveolin-1 gene expression, which may be closely related to intramuscular fatty infiltration. Thus, we selected Caveolin-1 as the study subject for the subsequent analysis.

### Caveolin-1 knockdown reduces fatty infiltration after RCT in rats

**Fig. 3:**
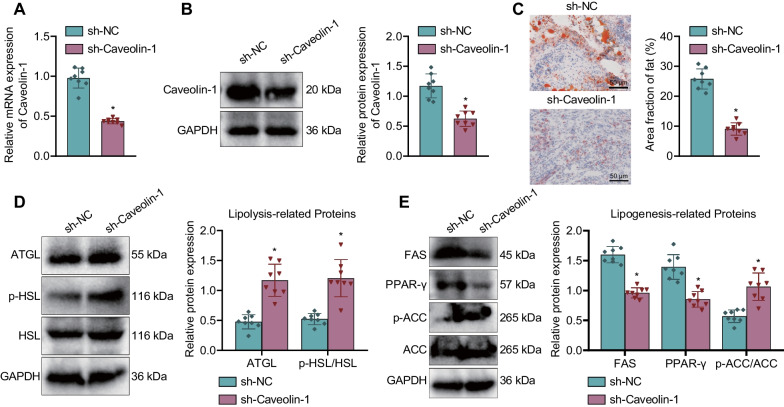
Caveolin-1 knockdown inhibits fatty infiltration after RCT in rats. **A** Caveolin-1 expression in the supraspinatus tendon of RCT rats treated with sh-Caveolin-1 detected by RT-qPCR. **B** Western blot of Caveolin-1 protein in the supraspinatus tendon of RCT rats treated with sh-Caveolin-1. **C** Fatty infiltration in the supraspinatus tendon of RCT rats treated with sh-Caveolin-1 evaluated by oil red O staining. **D** Western blot of lipolysis-related proteins (ATGL, pHSL/HSL) in the supraspinatus tendon of RCT rats treated with sh-Caveolin-1. **E** Western blot of adipogenesis-related proteins (FAS, PPAR-γ and pACC/ACC) in the supraspinatus tendon of RCT rats treated with sh-Caveolin-1. n = 8 rats for each treatment. *p < 0.05

Then we aimed to explore the effect of Caveolin-1 knockdown on fatty infiltration after RCT in rats. Caveolin-1 mRNA and protein expression was reduced in the supraspinatus tendon of RCT rats treated with sh-Caveolin-1 (Fig. 3A, B). The results of oil red O staining displayed a decline of fat area fraction in the supraspinatus tendon of RCT rats treated with sh-Caveolin-1 (Fig. 3C). Additionally, Western blot determination of lipolysis-related proteins (ATGL and pHSL/HSL) and lipogenesis-related proteins (FAS, PPAR-γ and pACC/ACC) unveiled an increase of ATGL expression and ratio of pHSL/HSL and pACC/ACC while a reduction was evident in FAS and PPAR-γ expression (Fig. 3D, E). The above results indicate that Caveolin-1 knockdown can significantly reduce fatty infiltration after RCT in rats.

### Caveolin-1 knockdown promotes bone-tendon healing, improves biomechanical properties, and reduces the rupture risk of injured tendon after RCT in rats

**Fig. 4:**
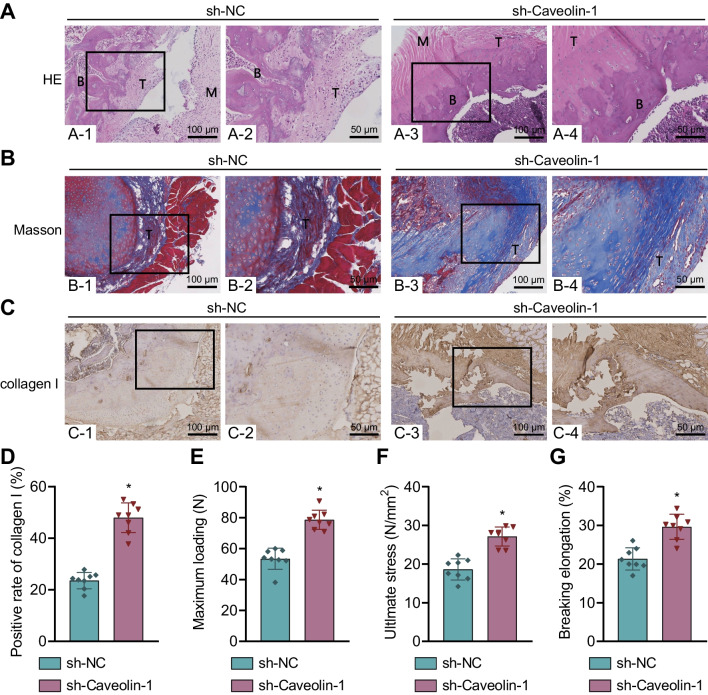
Caveolin-1 knockdown enhances bone-tendon healing, biomechanical properties, and inhibits rupture of injured tendon after RCTs in rats. **A** HE staining of the bone-tendon junction of sh-Caveolin-1-treated rats. **B** Shows the bone, M represents muscle, and T represents tendon. **B** Masson’s trichrome staining of the density of substrate and continuity of collagen fibers in sh-Caveolin-1-treated rats. **C** Immunohistochemistry of collagen I positive (brown) expression of sh-Caveolin-1-treated rats. **D** Quantitative analysis of panel **C**. **E** The maximum load of sh-Caveolin-1-treated rats measured by biomechanical test. **F** The limiting stress of sh-Caveolin-1-treated rats measured by biomechanical test. **G** The fracture elongation rate of sh-Caveolin-1-treated rats measured by biomechanical test. n = 8 rats for each treatment. *p < 0.05

The study subsequently centered on evaluating the effect of Caveolin-1 knockdown on bone-tendon healing, biomechanical properties, and rupture of injured tendon after RCT in rats. The results of HE staining, Masson’s trichrome staining and Immunohistochemistry revealed poor bone-tendon junction structure, low density of the substrate material, and weak continuity of the collagen fiber, lowered collagen I positive expression in the sh-NC-treated rats compared with the sham-operated rats. In contrast to the sh-NC-treated rats, the sh-Caveolin-1-treated rats exhibited opposite results (Fig. 4A–D). This suggests that knockdown of Caveolin-1 significantly promotes bone-tendon healing following RCT in rats.

Based on the biomechanical test results, the maximum load, limiting stress and fracture elongation rate were increased in the sh-Caveolin-1-treated rats (Fig. 4E–G). This suggests that knockdown of Caveolin-1 improves the biomechanical properties and reduces the rupture risk of injured tendon in rats.

Overall, knockdown of Caveolin-1 can facilitate bone-tendon healing following RCT, improve the biomechanical properties and decrease the rupture risk of injured tendon in rats.

### Caveolin-1 knockdown inhibits the adipogenic differentiation of TSCs through activation of the cAMP/PKA pathway

TSCs were isolated from rat Achilles tendon and the surface markers of TSCs were detected by flow cytometry. More than 97.5% of TSCs were found positive for CD44 and CD90, lower than 5.3% for CD34 and CD45, and 90.6% for CD146 (Additional file 1: Fig. S1A), consistent with a previous study (Bi et al. 2007). In addition, ARS and oil red O staining identified the osteogenic and adipogenic potential of TSCs (Additional file 1: Fig. S1B). These results indicate the successful isolation of the TSCs.

**Fig. 5:**
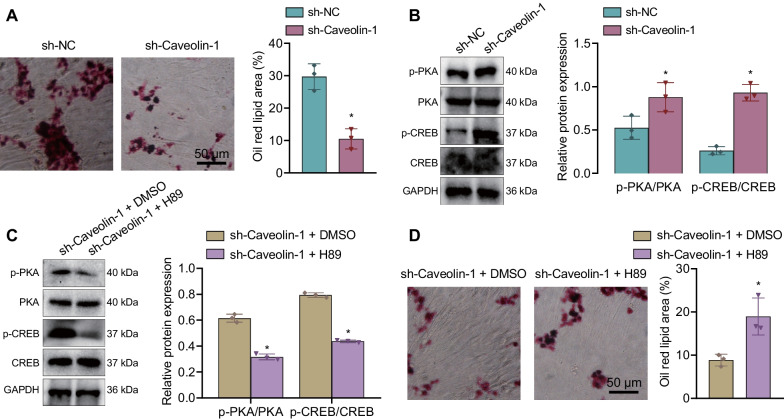
Caveolin-1 knockdown represses the adipogenic differentiation of TSCs via activation of the cAMP/PKA pathway. **A** Lipid droplet formation of TSCs treated with sh-Caveolin-1 observed by oil red O staining. **B** Western blot of the cAMP/PKA pathway-related proteins (pPKA, PKA, pCREB, and CREB) in TSCs treated with sh-Caveolin-1. **C** Western blot of the cAMP/PKA pathway-related proteins (pPKA, PKA, pCREB, and CREB) in TSCs treated with sh-Caveolin-1 or combined with H89. **D** Lipid droplet formation of TSCs treated with sh-Caveolin-1 or combined with H89 observed by oil red O staining. Cell experiments were repeated three times independently. *p < 0.05

RT-qPCR data showed downregulated Caveolin-1 expression in TSCs treated with sh-Caveolin-1#1, sh-Caveolin-1#2 or sh-Caveolin-1#3, with sh-Caveolin-1#1 showing the superior efficiency (Additional file 1: Fig. S1C) and selected for the subsequent studies. Oil red O staining results showed that silencing of Caveolin-1 in TSCs reduced lipid droplet formation (Fig. 5A). This indicates that the knockdown of Caveolin-1 inhibits the adipogenic differentiation of TSCs.

Moreover, Western blot results showed that silencing of Caveolin-1 augmented the ratio of pPKA/PKA and pCREB/CREB (Fig. 5B), demonstrating that Caveolin-1 may affect the adipogenic differentiation of TSCs by mediating the cAMP/PKA pathway. However, further treatment with H89 led to decreased ratio of pPKA/PKA and pCREB/CREB (Fig. 5C). According to oil red O staining data, lipid droplet formation was increased in response to treatment with sh-Caveolin-1 + H89 (Fig. 5D). The aforementioned results indicate that knockdown of Caveolin-1 inhibited the adipogenic differentiation of TSCs through activation of the cAMP/PKA pathway.

### GATA6, downregulated in the supraspinatus tendon, inhibits adipogenic differentiation of TSCs by diminishing Caveolin-1 expression

**Fig. 6:**
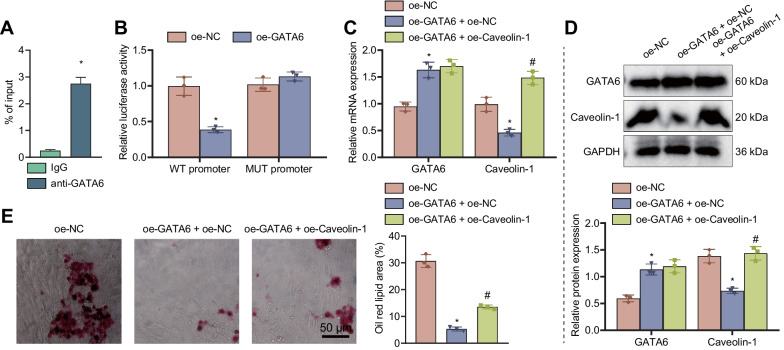
GATA6, downregulated in the supraspinatus tendon, inhibits adipogenic differentiation of TSCs by inhibiting Caveolin-1 expression. **A** The binding of GATA6 to the Caveolin-1 promoter region detected by ChIP assay. **B** The luciferase activity of Caveolin-1-WT and Caveolin-1-MUT in cells treated with oe-GATA6 determined by dual-luciferase reporter assay. **C** GATA6 and Caveolin-1 expression in TSCs treated with oe-GATA6 or combined with oe-Caveolin-1 detected by RT-qPCR. **D** Western blot of GATA6 and Caveolin-1 proteins in TSCs treated with oe-GATA6 or combined with oe-Caveolin-1. **E** ipid droplet formation of TSCs treated with oe-GATA6 or combined with oe-Caveolin-1 observed by oil red O staining. Cell experiments were repeated three times independently. *p < 0.05 compared with IgG, WT promoter, or oe-NC; # *p* < 0.05 compared with oe-GATA6 + oe-NC

Subsequently, the upstream mechanism by which Caveolin-1 affects the adipogenic differentiation of TSCs by mediating the cAMP/PKA pathway was the focus of this study. ChIP assay results indicated that GATA6 bound to the Caveolin-1 promoter region (Fig. 6A). Meanwhile, overexpression of GATA6 reduced the luciferase activity of the Caveolin-1 promoter (Fig. 6B). The results of RT-qPCR and Western blot demonstrated that GATA6 expression was increased and Caveolin-1 expression was decreased in TSCs treated with oe-GATA6 + oe-NC while oe-GATA6 + oe-Caveolin-1 did not alter GATA6 expression and elevated Caveolin-1 expression (Fig. 6C, D). Cumulatively, overexpression of GATA6 can inhibit the transcription of Caveolin-1 and repress its expression.

Furthermore, oil red O staining results showed a decrease in the lipid droplet formation upon GATA6 overexpression, the effect of which was negated following Caveolin-1 overexpression (Fig. 6E). The above results suggest that overexpression of GATA6 inhibited the adipogenic differentiation of TSCs by inhibiting Caveolin-1 expression.

### Overexpression of GATA6 inhibits Caveolin-1 expression and activates the cAMP/PKA pathway, thus reducing fatty infiltration after RCT in rats

**Fig. 7:**
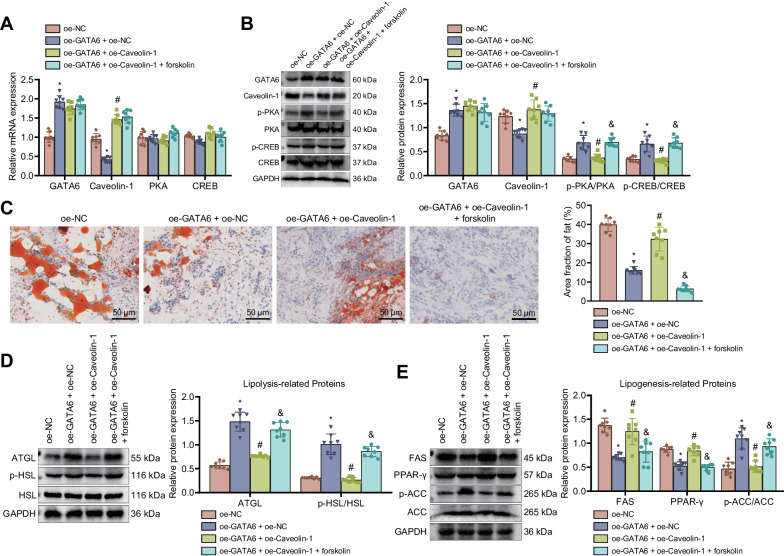
GATA6 suppresses fatty infiltration after RCT in rats by diminishing Caveolin-1 expression and activating the cAMP/PKA pathway. RCT rats were treated with oe-GATA6 + oe-NC, oe-GATA6 + oe-Caveolin-1 and oe-GATA6 + oe-Caveolin-1 + forskolin. **A** Expression of GATA6, Caveolin-1, PKA, and CREB in the supraspinatus tendon of RCT rats measured by RT-qPCR. **B** Western blot of GATA6, Caveolin-1, PKA, pPKA, CREB, and pCREB proteins in the supraspinatus tendon of RCT rats. **C** Fatty infiltration of supraspinatus tendon of RCT rats evaluated by oil red O staining. **D** Western blot of lipolysis-related proteins (ATGL and pHSL/HSL) in the supraspinatus tendon of RCT rats. **E** Western blot of adipogenesis-related proteins (FAS, PPAR-γPAand pACC/ACC) in the supraspinatus tendon of RCT rats. n = 8 rats for each treatment. *p < 0.05, compared with the RCT rats treated with oe-NC. #*p* < 0.05, compared with the RCT rats treated with oe-GATA6 + oe-NC. &*p* < 0.05, compared with the RCT rats treated with oe-GATA6 + oe-Caveolin-1

In this experiment, we attempted to validate the effect of GATA6 on fatty infiltration following RCT in rats by regulating Caveolin-1 expression and mediating the cAMP/PKA pathway. The results of RT-qPCR and Western blot suggested an enhancement of the mRNA and protein expression of GATA6 and the ratio of pPKA/PKA and pCREB/CREB yet a decline of the mRNA and protein expression of Caveolin-1 in the supraspinatus tendon of RCT rats treated with oe-GATA6 + oe-NC; however, opposite results were noted in response to treatment with oe-GATA6 + oe-Caveolin-1. In addition, the ratio of pPKA/PKA and pCREB/CREB was increased following treatment with oe-GATA6 + oe-Caveolin-1 + forskolin (Fig. 7A, B). This indicates that overexpression of GATA6 could activate the cAMP/PKA pathway by inhibiting Caveolin-1 expression.

As illustrated in Fig. 7C, overexpression of GATA6 decreased the fat area fraction, which was negated by overexpression of Caveolin-1. In addition, treatment with forskolin reduced the decreased fat area fraction. Western blot results showed elevated ATGL expression and increased ratio of pHSL/HSL yet diminished FAS and PPAR-γ-expression upon GATA6 overexpression whereas contrary results were found upon Caveolin-1 overexpression. Besides, forskolin treatment restored the effect of GATA6 overexpression (Fig. 7D, E).

Taken together, overexpression of GATA6 significantly reduced fatty infiltration after RCT in rats by diminishing Caveolin-1 expression and activating the cAMP/PKA pathway.

### GATA6 inhibits Caveolin-1 expression and activates the cAMP/PKA pathway, thus promoting bone-tendon healing, improving biomechanical properties, and reducing the rupture risk of injured tendon after RCT in rats

**Fig. 8:**
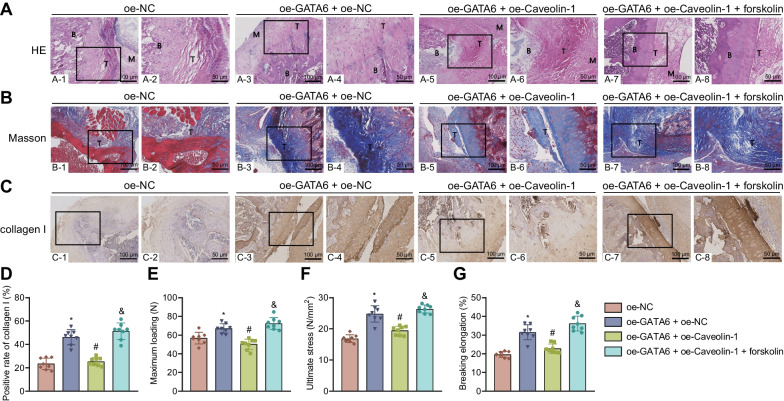
GATA6 regulates Caveolin-1/cAMP/PKA axis to promote bone-tendon healing, improve biomechanical properties, and reduce the rupture risk of injured tendon after RCT in rats. RCT rats were treated with oe-GATA6 + oe-NC, oe-GATA6 + oe-Caveolin-1 and oe-GATA6 + oe-Caveolin-1 + forskolin. **A** HE staining of the bone-tendon junction of RCT rats. **B** Shows the bone, M represents muscle, and T represents tendon. **B** Masson’s trichrome staining of the density of substrate and continuity of collagen fibers in RCT rats. **C** Immunohistochemistry of collagen I positive (brown) expression of RCT rats. **D** Quantitative analysis of panel **C**. **E** The maximum load of RCT rats measured by biomechanical test. **F** The limiting stress of RCT rats measured by biomechanical test. **G** The fracture elongation rate of RCT rats measured by biomechanical test. n = 8 rats for each treatment. *p < 0.05, compared with the RCT rats treated with oe-NC. #*p* < 0.05, compared with the RCT rats treated with oe-GATA6 + oe-NC. &*p* < 0.05, compared with the RCT rats treated with oe-GATA6 + oe-Caveolin-1

At last, we sought to verify the effect of GATA6 on bone-tendon healing, biomechanical properties and rupture of injured tendon after RCT in rats by regulating Caveolin-1 expression and mediating the cAMP/PKA pathway. Based on the results of HE staining, Masson’s trichrome staining and Immunohistochemistry, there appeared orderly bone-tendon junction structure, high density of the substrate material, and strong continuity of the collagen fiber, and increased collagen I positive expression in the presence of abundantly expressed GATA6. In contrast, abundantly expressed Caveolin-1 led to opposite results. Further treatment with forskolin restored the effect of abundantly expressed GATA6 (Fig. 8A–D). This suggests that GATA6 overexpression activates the cAMP/PKA pathway by inhibiting Caveolin-1 expression and subsequently promotes bone-tendon healing after RCT in rats.

The biomechanical test results displayed that ectopically expressed GATA6 increased the maximum load, limiting stress and fracture elongation rate, which were opposite following abundantly expressed Caveolin-1. Furthermore, treatment with forskolin restored the effect of ectopically expressed GATA6 (Fig. 8E–G).

These data reveal that overexpression of GATA6 can activate the cAMP/PKA pathway by inhibiting Caveolin-1 expression, thereby enhancing the biomechanical properties and reducing the rupture risk of injured tendon after RCT in rats.

## Discussion

In this study, we investigated the upstream and downstream mechanism of Caveolin-1 on the fatty infiltration and bone-tendon healing following RCT. The results showed that GATA6 can potentially reduce fatty infiltration, enhance bone-tendon healing, enhance biomechanical properties, and decrease the rupture risk of injured tendon after RCT, which was associated with activated cAMP/PKA pathway and diminished Caveolin-1 expression.

The obtained data showed that Caveolin-1 may play a role in RCT process and is related to fatty infiltration. ATGL and pHSL/HSL are known as markers of lipolysis and their upregulated levels indicate reduced fatty infiltration (Stofkova et al. 2016; Chen et al. 2021). In addition, FAS, PPAR-γ and pACC/ACC are lipogenesis-related proteins; increased PPAR-γ expression and diminished FAS and ACC expression reflect inhibited fatty infiltration (Lv et al. 2018; Do et al. 2019). In this study, the results suggested that Caveolin-1 knockdown resulted in an increase of ATGL expression and ratio of pHSL/HSL and pACC/ACC while a reduction was evident in FAS and PPAR-γ expression. Therefore, based on the previous reports and the current results, it can be plausible that Caveolin-1 knockdown could reduce fatty infiltration after RCT.

The subsequent results of this study revealed that Caveolin-1 knockdown could promote bone-tendon healing, improve biomechanical properties, and reduce the rupture risk of injured tendon after RCT in rats. In accordance with this finding, a recent work has demonstrated that **C**aveolin-1 can enhance biomechanical properties and accelerate bone-tendon junction healing by promoting the formation of the transition zone (Liang et al. 2019). In addition, the in vitro data suggested that Caveolin-1 knockdown suppressed the adipogenic differentiation of TSCs via activation of the cAMP/PKA pathway. Indeed, previously published literature has demonstrated that Caveolin-1 secreted by adipose tissues and adipocytes can promote adipogenesis (Chang et al. 2017). Also, Caveolin-1 can promote the lipid droplet formation (Cohen et al. 2004). The activity of PKA is found greatly increased in adipocytes upon Caveolin-1 knockdown (Cohen et al. 2004). In addition, the absence of Caveolin-1 has been reported to activate the cAMP/PKA signaling to inhibit lipid droplet formation in endothelial cells (Kuo et al. 2018). A recent study has reported the suppressing effect of activated cAMP/PKA pathway on the adipogenic differentiation of MC3T3E1 cells (Ma et al. 2021). However, the inhibiting property of the cAMP/PKA pathway on the adipogenic differentiation of TSCs lacks available evidence to support and requires further studies. The tendinopathy can be caused by the aberrant differentiation of TSCs and the inhibition of the aberrant differentiation of TSCs is thus a major target for the regeneration of damaged tendon tissues (Kim et al. 2020). Notably, the enhanced cAMP concentration aids in the treatment of Achilles tendon injury in rats (Rho et al. 2020). Thus, targeting the Caveolin-1/cAMP/PKA axis may enable development of novel targets for treatment of bone-tendon injury following RCT.

Furthermore, this study revealed that GATA6 can inhibit Caveolin-1 expression and activated the cAMP/PKA pathway. Consistently, GATA6 binds to the promoter of human and mouse Caveolin-1 gene, contributing to reduced expression of Caveolin-1 (Boopathi et al. 2011). In addition, a previous study has highlighted that cAMP/PKA pathway can stimulate GATA6 gene expression (Tremblay et al. 2003), which is opposite to our results and the further investigation is required for validation. Furthermore, our findings further provide evidence for supporting the development and application of anti-Caveolin-1 therapy for RCT patients in clinical setting, including the development and testing of chemical small molecule targeted inhibitors and gene targeted drugs. This may improve the treatment outcomes of RCT-associated diseases and reduce the adverse events of steroid-like drugs or anti-inflammatory drugs.

## Conclusion

**Fig. 9:**
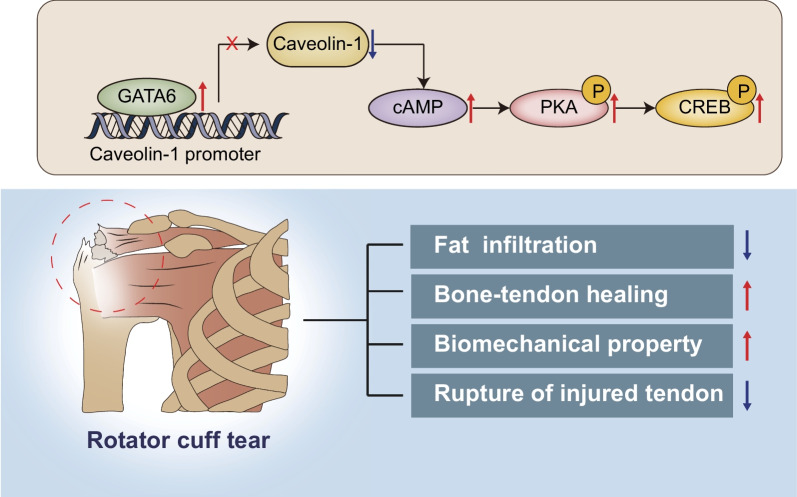
Schematic representation summarizing the role of GATA6 in RCT. GATA6 activates the cAMP/PKA pathway by inhibiting Caveolin-1 expression, thus reducing fatty infiltration, promoting bone-tendon healing, improving biomechanical properties, and decreasing the rupture risk of injured tendon after RCT

In summary, the results of this study suggest that GATA6 can inhibit Caveolin-1 expression and activate the cAMP/PKA pathway, therefore inhibiting fatty infiltration, accelerating bone-tendon healing, improving biomechanical properties, and reducing the rupture risk of injured tendon after RCT in rats (Fig. 9). These findings provide mechanistic understanding for further investigation aimed at clarifying the pathogenesis of RCT and identifying promising therapeutic targets. Nonetheless, available evidence is limited regarding the role of GATA6 in the fatty infiltration, adipogenic differentiation of TSCs and bone-tendon healing. Thus, more in-depth investigation is still warranted.

## Supplementary Information

**Additional file 1:** **Fig. S1** Identification of TSCs and Caveolin-1 knockdown efficiency. A, Flow cytometric analysis of TSC-related markers (CD44, CD90, CD146, CD34 and CD45). B, Matrix mineralization determined by ARS staining, and lipid droplet formation determined by oil red O staining. C, Knockdown efficiency of sh-Caveolin-1#1, sh-Caveolin-1#2 and sh-Caveolin-1#3 in TSCs determined by RT-qPCR. Cell experiments were repeated three times independently.

**Additional file 2**: **Table S1** shRNA sequences. **Table S2 **Primer sequences for RT-qPCR. 

## Data Availability

The data underlying this article will be shared on reasonable request to the corresponding author.

## Abbreviations

RCT: Rotator cuff tear
TSCs: Tendon stem cells
GATA6: GATA binding protein 6
DEGs: Differentially expressed genes
