# Supplementary material for: Caveolin-1 is involved in fatty infiltration and bone-tendon healing of rotator cuff tear
Source: Mol Med. 2023 Mar 14;29:33. doi: 10.1186/s10020-023-00627-4 (PMC10015686; doi:10.1186/s10020-023-00627-4)
Supplement: Supplementary file 2 — Additional file 2: Table S1 shRNA sequences. Table S2 Primer sequences for RT-qPCR. [file 10020_2023_627_MOESM2_ESM.docx]

**Table S1** shRNA sequences

| shRNA | Sequence (5’-3’) |
| --- | --- |
| sh-Caveolin-1#1 (rat) | GGCATCTACTTTGCCATCCTCTCTT |
| sh-Caveolin-1#2 (rat) | CCGTGATTTCTGAATGGTTTGTCTT |
| sh-Caveolin-1#3 (rat) | CCCGTTCCTTTGTGGATCATTGTTT |

**Table S2** Primer sequences for RT-qPCR

| Gene | Sequence (5’-3’) |
| --- | --- |
| GATA6 (rat) | Forward: GCCAACTGTCACACCACAAC  Reverse: CAGACAAGGCCCCAGTCATT |
| Caveolin-1 (rat) | Forward: GCGCCTTTCCCCCTCTATAC  Reverse: AGATGCCGTCGAAACTGTGT |
| PKA (rat) | Forward: GAGCAGGAGAGCGTGAAAGA  Reverse: TCCTTGTGCTTCACGAGCAT |
| CREB (rat) | Forward: GACGGAGGAGCTTGTACCAC  Reverse: GCACTGCCACTCTGTTCTCT |
| GAPDH (rat) | Forward: GCATCTTCTTGTGCAGTGCC  Reverse: GATGGTGATGGGTTTCCCGT |
